# White’s Toothache Drops

**Published:** 1848-10

**Authors:** 


### WHITE'S TOOTH-ACHE DROPS.

As early as 1829, and many years subsequently, a secret remedy for the cure of toothache, and also for the prevention and cure of every variety of disease to which the teeth are liable, by applying the nostrum to their surfaces, obtained extensive sale throughout the United States. It was denominated "White's tooth-ache drops," and was recommended by certificates from Dental Surgeons and others who had been made acquainted with its composition. The Recipe fell into our hands, accidentally, during our professional career in Philadelphia;—here it is *verbatim et literatim.*

"Resepee for making White's tooth-ache drops. Take one oz. of strong tincter of Opeom, and do of strong camphorated spirits of wine, and 1 oz of oil of peppermint, mix them together then add about one half oz of Nitric Acid if it is not two strong."

(Signed) T. WHITE.

Vast mischief has been done by the use of the above combination, and, especially, when applied to the teeth as a preservative against decay. Indeed, I have seen hundreds of teeth ruined by its use, and yet, perhaps, much of the mischief which it effected was through the recommendation of those who should have known better, and whose duty it was to protect the unsuspecting against imposition, and the profession against the imputation of ignorance and quackery.—ST. LOUIS ED.

